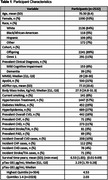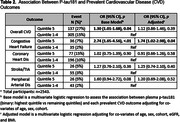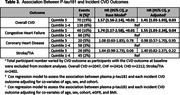# The Association Between Plasma P‐tau181 and Cardiovascular Disease in the Framingham Heart Study

**DOI:** 10.1002/alz.093315

**Published:** 2025-01-09

**Authors:** Jeremy A. Tanner, Crystal Wiedner, Hugo J. Aparicio, Mitzi M. Gonzales, Dibya Himali, Tiffany F. Kautz, Emer R McGrath, Alicia S. Parker, Jaime Ramos‐Cejudo, Claudia L Satizabal, Alexa S Beiser, Russell P. Tracy, Ramachandran S Vasan, Jayandra J. Himali, Sudha Seshadri

**Affiliations:** ^1^ Glenn Biggs Institute for Alzheimer’s & Neurodegenerative Diseases, University of Texas Health Science Center, San Antonio, TX USA; ^2^ Boston University School of Medicine, Boston, MA USA; ^3^ Cedars‐Sinai Medical Center, Los Angeles, CA USA; ^4^ HRB Clinical Research Facility, National University of Ireland Galway, Galway Ireland; ^5^ Glenn Biggs Institute for Alzheimer’s & Neurodegenerative Diseases, San Antonio, TX USA; ^6^ NYU School of Medicine, New York, NY USA; ^7^ Glenn Biggs Institute for Alzheimer’s & Neurodegenerative Diseases, University of Texas Health Science Center at San Antonio, San Antonio, TX USA; ^8^ University of Vermont, Colchester, VT USA; ^9^ The University of Texas School of Public Health San Antonio, San Antonio, TX USA

## Abstract

**Background:**

Plasma p‐tau biomarkers are promising diagnostic tools for widespread clinical use. However, recent studies have raised concerns regarding the effect of common medical comorbidities, such as cardiovascular disease (CVD), on plasma p‐tau specificity. These influences must be better understood to enable appropriate clinical use of p‐tau181. We sought to evaluate the association between p‐tau181 and CVD outcomes in the Framingham Heart Study (FHS), a population‐based prospective cohort with deep‐phenotyping of CVD.

**Method:**

FHS Offspring and Omni 1 Cohort participants have been followed through quadrennial exams with detailed CVD assessment. Plasma p‐tau181 was measured from samples of 2543 participants collected in 2011‐2014 using Quanterix Simoa, and analyzed as a binary predictor (highest quintile vs remainder). CVD outcomes (binary:yes/no) included overall CVD, congestive heart failure(CHF), coronary heart disease(CHD), stroke/TIA, and peripheral arterial disease(PAD). Multivariate logistic regressions were performed to assess the association between p‐tau181 and each prevalent CVD outcome adjusting for age, sex, cohort in base models, and additionally for eGFR, and BMI in adjusted models. Cox regression models were performed to assess the association between p‐tau181 and incident CVD outcomes adjusting for similar covariates after a median survival time of 7.0 years [5.9‐7.6]).

**Result:**

Participant characteristics are displayed in Table 1. Elevated p‐tau181 was associated with 74% higher odds of prevalent CHF (OR 1.74; 95%CI[1.02‐2.98], p=0.04) in adjusted models (Table 2). Elevated p‐tau181 was associated with 41% higher risk of incident overall CVD (HR 1.41[1.03‐1.93], p=0.03) and 55% higher risk of incident CHF (1.55[1.03‐2.34], p=0.04) in adjusted models (Table 3). There was no association between p‐tau181 levels and other CVD outcomes in cross‐sectional or longitudinal analyses.

**Conclusion:**

Elevated plasma p‐tau181 is associated with prevalent and incident CHF and with overall CVD in a community‐based population. This aligns with growing evidence of a possible bidirectional relationship between CHF and AD. P‐tau levels should be interpreted with caution in patients with CHF until the link between p‐tau181 and CHF can be further clarified. Studies to understand systemic diseases that influence plasma AD biomarkers are necessary prior to widespread clinical use, and can reveal new relationships between AD and systemic diseases.